# 3-(4-Fluoro­benz­yl)-1*H*-isochromene-1-thione

**DOI:** 10.1107/S1600536809004152

**Published:** 2009-02-11

**Authors:** Tariq Mahmood Babar, Ghulam Qadeer, Nasim Hasan Rama, Muhammad Khawar Rauf, Wai-Yeung Wong

**Affiliations:** a Department of Chemistry, Quaid-i-azam University, Islamabad 45320, Pakistan; b Department of Chemistry, Hong Kong Baptist University, Waterloo Road, Kowloon Tong, Hong Kong, People’s Republic of China

## Abstract

In the mol­ecule of the title compound, C_16_H_11_FOS, the benzene ring is oriented at a dihedral angle of 89.68 (3)° with respect to the planar [maximum deviation 0.009 (2) Å]  isocoumarin ring system. An intra­molecular C—H⋯S inter­action results in the formation of a planar five-membered ring. In the crystal structure, inter­molecular C—H⋯O hydrogen bonds link the mol­ecules into chains parallel to the *c* axis. A π–π contact between the isocoumarin rings [centroid–centroid distance = 3.818 (3) Å] may further stabilize the structure.

## Related literature

For general background, see: Barry (1964 ▶); Sturtz *et al.* (2002 ▶); Rossi *et al.* (2003 ▶); Powers *et al.* (2002 ▶); Thomas & Jens (1999 ▶). For a related structure, see: Abid *et al.* (2006 ▶). For bond-length data, see: Allen *et al.* (1987 ▶).
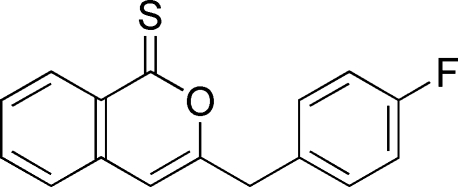


## Experimental

### 

#### Crystal data

C_16_H_11_FOS
*M* *_r_* = 270.31
Monoclinic, 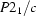

*a* = 8.7346 (6) Å
*b* = 17.9516 (11) Å
*c* = 8.4481 (5) Å
β = 95.026 (1)°
*V* = 1319.57 (14) Å^3^
*Z* = 4
Mo *K*α radiation
μ = 0.25 mm^−1^
*T* = 294 (2) K
0.30 × 0.25 × 0.20 mm

#### Data collection

Bruker SMART CCD area-detector diffractometer
Absorption correction: multi-scan (*SADABS*; Bruker, 2001 ▶) *T*_min_ = 0.805, *T*_max_ = 0.952
7856 measured reflections
3188 independent reflections
2655 reflections with *I* > 2σ(*I*)
*R*_int_ = 0.016

#### Refinement

*R*[*F* ^2^ > 2σ(*F* ^2^)] = 0.046
*wR*(*F* ^2^) = 0.150
*S* = 1.03
3188 reflections
172 parameters
H-atom parameters constrained
Δρ_max_ = 0.34 e Å^−3^
Δρ_min_ = −0.30 e Å^−3^

### 

Data collection: *SMART* (Bruker, 2001 ▶); cell refinement: *SAINT* (Bruker, 2002 ▶); data reduction: *SAINT*; program(s) used to solve structure: *SHELXS97* (Sheldrick, 2008 ▶); program(s) used to refine structure: *SHELXL97* (Sheldrick, 2008 ▶); molecular graphics: *ORTEP-3 for Windows* (Farrugia, 1997 ▶) and *PLATON* (Spek, 2003 ▶); software used to prepare material for publication: *SHELXTL* (Sheldrick, 2008 ▶) and *PLATON*.

## Supplementary Material

Crystal structure: contains datablocks I, global. DOI: 10.1107/S1600536809004152/hk2617sup1.cif

Structure factors: contains datablocks I. DOI: 10.1107/S1600536809004152/hk2617Isup2.hkl

Additional supplementary materials:  crystallographic information; 3D view; checkCIF report

## Figures and Tables

**Table 1:** Hydrogen-bond geometry (Å, °)

*D*—H⋯*A*	*D*—H	H⋯*A*	*D*⋯*A*	*D*—H⋯*A*
C1—H1*A*⋯S1	0.93	2.78	3.142 (2)	105
C3—H3*A*⋯O1^i^	0.93	2.60	3.529 (2)	178

Symmetry code: (i) 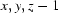.

**Fig. 1.**
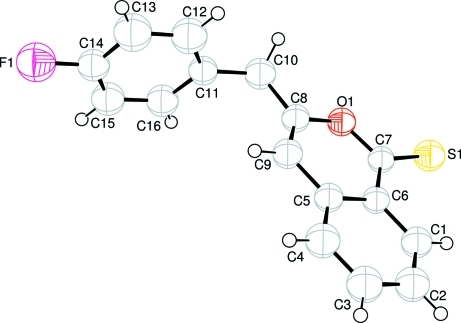
The molecular structure of the title molecule, with the atom-numbering scheme.

**Fig. 2.**
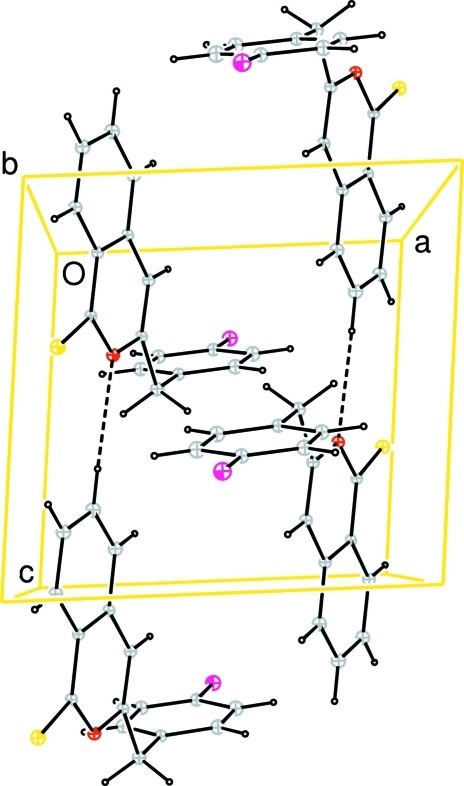
A partial packing diagram of the title compound. Hydrogen bonds are shown as dashed lines.

**Fig. 3.**
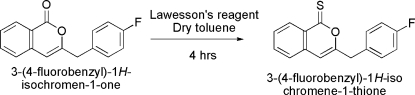
The formation of the title compound.

## supplementary crystallographic information

### Comment

The isocoumarin nucleus is an abundant structural motif in natural products (Barry, 1964). Many constituents of the steadily growing class of known isocoumarins exhibit valuable biological properties such as antifungal (Sturtz *et al.*,  2002), antitumor or cytotoxic, anti-inflammatory, anti-allergic (Rossi *et al.*,  2003) and enzyme inhibitory  (Powers *et al.*,  2002) activities. Naturally occurring halo-isocoumarins and their halogeno-3,4-dihydroiscoumarin derivatives are very rare. However, a few examples of naturally occurring chlorine containing isocoumarins are known (Thomas & Jens, 1999). In view of the importance of this class of compounds, the title compound, an isocoumarine derivative containing 4-fluorobenzyl substituent has been synthesized, and we report herein its crystal structure.

In the molecule of the title compound (Fig. 1), the bond lengths (Allen *et al.*,  1987) and angles are within normal ranges, and comparable with the corresponding values in 3-(2-chlorobenzyl)isocoumarin (Abid *et al.*, 2006). Rings A (C1-C6), B (O1/C5-C9) and C (C11-C16) are, of course, planar and the dihedral angles between them are A/B = 0.29 (3)°, A/C = 89.77 (4)° and B/C = 89.53 (3)°. The intramolecular C-H···S interaction (Table 1) results in the formation of a planar five-membered ring D (S1/C1/C6/C7/H1A).

In the crystal structure, intermolecular C-H···O hydrogen bonds (Table 1) link the molecules into chains parallel to the c-axis (Fig. 2), in which they may be effective in the stabilization of the structure. The π-π contact between the isocoumarine rings, Cg1—Cg2^i^ [symmetry code: (i) -x, -y, 1 - z, where Cg1 and Cg2 are centroids of the rings A (C1-C6) and B (O1/C5-C9), respectively] may further stabilize the structure, with centroid-centroid distance of 3.818 (3) Å.

### Experimental

As shown in Scheme 2, the title compound was synthesized by refluxing 3-(4-fluorobenzyl)-1*H*-isochromen-1-one (0.5 g, 1.8 mmol) with Lawesson's reagent (0.89 g, 2.2 mmol) in dry toluene for 4 h. Pure thioisocoumarin was obtained by recrystalization in methanol (yield; 90%, m.p. 665-667 K).

### Refinement

H atoms were positioned geometrically, with C-H = 0.93 and 0.97 Å for aromatic and methylene H, respectively, and constrained to ride on their parent atoms, with U_iso_(H) = 1.2U_eq_(C).

### Crystal data

**Table:**

C_16_H_11_FOS	*F*(000) = 560
*M**_r_* = 270.31	*D*_x_ = 1.361 Mg m^−^^3^
Monoclinic, *P*2_1_/*c*	Melting point: 392(2) K
Hall symbol: -P 2ybc	Mo *K*α radiation, λ = 0.71073 Å
*a* = 8.7346 (6) Å	Cell parameters from 1423 reflections
*b* = 17.9516 (11) Å	θ = 4.2–25.4°
*c* = 8.4481 (5) Å	µ = 0.25 mm^−^^1^
β = 95.026 (1)°	*T* = 294 K
*V* = 1319.57 (14) Å^3^	Block, yellow
*Z* = 4	0.30 × 0.25 × 0.20 mm

### Data collection

**Table:**

Bruker SMART CCD area-detector diffractometer	3188 independent reflections
Radiation source: fine-focus sealed tube	2655 reflections with *I* > 2σ(*I*)
graphite	*R*_int_ = 0.016
φ and ω scans	θ_max_ = 28.3°, θ_min_ = 2.6°
Absorption correction: multi-scan (*SADABS*; Bruker, 2001)	*h* = −11→11
*T*_min_ = 0.805, *T*_max_ = 0.952	*k* = −23→22
7856 measured reflections	*l* = −8→11

### Refinement

**Table:**

Refinement on *F*^2^	Primary atom site location: structure-invariant direct methods
Least-squares matrix: full	Secondary atom site location: difference Fourier map
*R*[*F*^2^ > 2σ(*F*^2^)] = 0.046	Hydrogen site location: inferred from neighbouring sites
*wR*(*F*^2^) = 0.150	H-atom parameters constrained
*S* = 1.03	*w* = 1/[σ^2^(*F*_o_^2^) + (0.0856*P*)^2^ + 0.2983*P*] where *P* = (*F*_o_^2^ + 2*F*_c_^2^)/3
3188 reflections	(Δ/σ)_max_ < 0.001
172 parameters	Δρ_max_ = 0.34 e Å^−^^3^
0 restraints	Δρ_min_ = −0.29 e Å^−^^3^

### Special details

**Table:**

Geometry. All e.s.d.'s (except the e.s.d. in the dihedral angle between two l.s. planes) are estimated using the full covariance matrix. The cell e.s.d.'s are taken into account individually in the estimation of e.s.d.'s in distances, angles and torsion angles; correlations between e.s.d.'s in cell parameters are only used when they are defined by crystal symmetry. An approximate (isotropic) treatment of cell e.s.d.'s is used for estimating e.s.d.'s involving l.s. planes.
Refinement. Refinement of *F*^2^ against ALL reflections. The weighted *R*-factor *wR* and goodness of fit *S* are based on *F*^2^, conventional *R*-factors *R* are based on *F*, with *F* set to zero for negative *F*^2^. The threshold expression of *F*^2^ > σ(*F*^2^) is used only for calculating *R*-factors(gt) *etc*. and is not relevant to the choice of reflections for refinement. *R*-factors based on *F*^2^ are statistically about twice as large as those based on *F*, and *R*- factors based on ALL data will be even larger.

### Fractional atomic coordinates and isotropic or equivalent isotropic displacement parameters (Å^2^)

**Table:**

	*x*	*y*	*z*	*U*_iso_*/*U*_eq_	
S1	0.07012 (6)	0.65948 (3)	0.36335 (6)	0.0646 (2)	
O1	0.20272 (15)	0.53300 (6)	0.37410 (13)	0.0506 (3)	
F1	0.51024 (16)	0.13358 (7)	0.29180 (19)	0.0845 (4)	
C1	0.0938 (2)	0.63191 (10)	−0.0010 (2)	0.0563 (4)	
H1A	0.0472	0.6745	0.0350	0.068*	
C2	0.1045 (3)	0.62201 (12)	−0.1609 (2)	0.0665 (5)	
H2A	0.0656	0.6581	−0.2326	0.080*	
C3	0.1727 (3)	0.55866 (12)	−0.2156 (2)	0.0706 (6)	
H3A	0.1785	0.5521	−0.3241	0.085*	
C4	0.2320 (3)	0.50543 (11)	−0.1113 (2)	0.0656 (5)	
H4A	0.2784	0.4633	−0.1496	0.079*	
C5	0.2234 (2)	0.51390 (9)	0.05279 (18)	0.0483 (4)	
C6	0.15271 (18)	0.57814 (8)	0.10781 (17)	0.0439 (3)	
C7	0.14344 (18)	0.58791 (9)	0.27669 (18)	0.0446 (3)	
C8	0.2707 (2)	0.46929 (8)	0.32005 (19)	0.0482 (4)	
C9	0.2829 (2)	0.45929 (9)	0.1661 (2)	0.0523 (4)	
H9A	0.3304	0.4166	0.1313	0.063*	
C10	0.3182 (3)	0.41971 (10)	0.4583 (2)	0.0615 (5)	
H10A	0.2320	0.4141	0.5222	0.074*	
H10B	0.4006	0.4438	0.5237	0.074*	
C11	0.3716 (2)	0.34333 (9)	0.4123 (2)	0.0515 (4)	
C12	0.5239 (3)	0.32831 (12)	0.3957 (3)	0.0741 (6)	
H12A	0.5961	0.3662	0.4129	0.089*	
C13	0.5718 (2)	0.25794 (14)	0.3540 (3)	0.0799 (7)	
H13A	0.6748	0.2483	0.3421	0.096*	
C14	0.4648 (2)	0.20356 (10)	0.3309 (2)	0.0585 (4)	
C15	0.3136 (2)	0.21543 (11)	0.3461 (3)	0.0671 (5)	
H15A	0.2425	0.1771	0.3292	0.081*	
C16	0.2677 (2)	0.28622 (11)	0.3875 (3)	0.0644 (5)	
H16A	0.1644	0.2952	0.3987	0.077*	

### Atomic displacement parameters (Å^2^)

**Table:**

	*U*^11^	*U*^22^	*U*^33^	*U*^12^	*U*^13^	*U*^23^
S1	0.0761 (4)	0.0604 (3)	0.0583 (3)	0.0200 (2)	0.0112 (2)	−0.0054 (2)
O1	0.0702 (8)	0.0431 (6)	0.0390 (5)	0.0077 (5)	0.0071 (5)	−0.0006 (4)
F1	0.0826 (9)	0.0557 (7)	0.1132 (11)	0.0188 (6)	−0.0030 (8)	−0.0146 (7)
C1	0.0629 (11)	0.0529 (9)	0.0526 (9)	0.0022 (8)	0.0028 (7)	0.0071 (7)
C2	0.0840 (14)	0.0661 (11)	0.0485 (9)	−0.0040 (10)	−0.0003 (9)	0.0157 (9)
C3	0.1044 (17)	0.0684 (12)	0.0396 (8)	−0.0116 (11)	0.0106 (9)	0.0040 (8)
C4	0.1026 (16)	0.0520 (9)	0.0441 (9)	−0.0023 (10)	0.0173 (9)	−0.0040 (7)
C5	0.0635 (10)	0.0415 (7)	0.0408 (7)	−0.0063 (7)	0.0093 (7)	−0.0009 (6)
C6	0.0473 (8)	0.0431 (7)	0.0412 (7)	−0.0052 (6)	0.0042 (6)	0.0012 (6)
C7	0.0462 (8)	0.0438 (7)	0.0437 (7)	−0.0001 (6)	0.0043 (6)	0.0000 (6)
C8	0.0623 (10)	0.0364 (7)	0.0460 (8)	0.0016 (6)	0.0053 (7)	−0.0004 (6)
C9	0.0713 (11)	0.0383 (7)	0.0484 (8)	0.0032 (7)	0.0110 (7)	−0.0020 (6)
C10	0.0910 (14)	0.0465 (9)	0.0466 (9)	0.0090 (9)	0.0037 (8)	0.0035 (7)
C11	0.0653 (10)	0.0424 (8)	0.0464 (8)	0.0041 (7)	0.0019 (7)	0.0069 (6)
C12	0.0598 (12)	0.0560 (11)	0.1061 (18)	−0.0107 (9)	0.0047 (11)	−0.0067 (11)
C13	0.0506 (11)	0.0685 (13)	0.121 (2)	0.0047 (9)	0.0111 (12)	−0.0073 (13)
C14	0.0621 (11)	0.0453 (8)	0.0666 (11)	0.0093 (7)	−0.0027 (8)	−0.0016 (8)
C15	0.0561 (11)	0.0462 (9)	0.0976 (15)	−0.0034 (8)	−0.0018 (10)	−0.0022 (9)
C16	0.0508 (10)	0.0538 (10)	0.0891 (14)	0.0065 (8)	0.0080 (9)	0.0029 (9)

### Geometric parameters (Å, °)

**Table:**

C1—C2	1.373 (3)	C8—C10	1.498 (2)
C1—C6	1.400 (2)	C9—H9A	0.9300
C1—H1A	0.9300	C10—C11	1.510 (2)
C2—C3	1.382 (3)	C10—H10A	0.9700
C2—H2A	0.9300	C10—H10B	0.9700
C3—C4	1.370 (3)	C11—C16	1.373 (3)
C3—H3A	0.9300	C11—C12	1.376 (3)
C4—C5	1.403 (2)	C12—C13	1.386 (3)
C4—H4A	0.9300	C12—H12A	0.9300
C5—C6	1.406 (2)	C13—C14	1.353 (3)
C5—C9	1.435 (2)	C13—H13A	0.9300
C6—C7	1.447 (2)	C14—C15	1.355 (3)
C7—O1	1.3573 (19)	C14—F1	1.367 (2)
C7—S1	1.6367 (16)	C15—C16	1.387 (3)
C8—C9	1.327 (2)	C15—H15A	0.9300
C8—O1	1.3843 (18)	C16—H16A	0.9300
			
C2—C1—C6	120.26 (18)	C5—C9—H9A	119.8
C2—C1—H1A	119.9	C8—C10—C11	114.22 (15)
C6—C1—H1A	119.9	C8—C10—H10A	108.7
C1—C2—C3	120.26 (18)	C11—C10—H10A	108.7
C1—C2—H2A	119.9	C8—C10—H10B	108.7
C3—C2—H2A	119.9	C11—C10—H10B	108.7
C4—C3—C2	120.55 (17)	H10A—C10—H10B	107.6
C4—C3—H3A	119.7	C16—C11—C12	118.03 (17)
C2—C3—H3A	119.7	C16—C11—C10	120.16 (18)
C3—C4—C5	120.66 (18)	C12—C11—C10	121.81 (18)
C3—C4—H4A	119.7	C11—C12—C13	121.35 (19)
C5—C4—H4A	119.7	C11—C12—H12A	119.3
C4—C5—C6	118.58 (15)	C13—C12—H12A	119.3
C4—C5—C9	122.49 (16)	C14—C13—C12	118.3 (2)
C6—C5—C9	118.92 (14)	C14—C13—H13A	120.8
C1—C6—C5	119.68 (15)	C12—C13—H13A	120.8
C1—C6—C7	121.00 (15)	C13—C14—C15	122.61 (18)
C5—C6—C7	119.31 (14)	C13—C14—F1	119.12 (18)
O1—C7—C6	117.29 (13)	C15—C14—F1	118.27 (18)
O1—C7—S1	116.25 (11)	C14—C15—C16	118.29 (18)
C6—C7—S1	126.45 (12)	C14—C15—H15A	120.9
C9—C8—O1	120.60 (14)	C16—C15—H15A	120.9
C9—C8—C10	130.03 (16)	C11—C16—C15	121.39 (18)
O1—C8—C10	109.36 (13)	C11—C16—H16A	119.3
C8—C9—C5	120.38 (15)	C15—C16—H16A	119.3
C8—C9—H9A	119.8	C7—O1—C8	123.49 (12)

### Hydrogen-bond geometry (Å, °)

**Table:**

*D*—H···*A*	*D*—H	H···*A*	*D*···*A*	*D*—H···*A*
C1—H1A···S1	0.93	2.78	3.142 (2)	105
C3—H3A···O1^i^	0.93	2.60	3.529 (2)	178

Symmetry codes: (i) *x*, *y*, *z*−1.
